# From Acidifiers to Intestinal Health Enhancers: How Organic Acids Can Improve Growth Efficiency of Pigs

**DOI:** 10.3390/ani10010134

**Published:** 2020-01-14

**Authors:** Benedetta Tugnoli, Giulia Giovagnoni, Andrea Piva, Ester Grilli

**Affiliations:** 1 Vetagro S.p.A.-Via Porro 2, 42124 Reggio Emilia, Italy; benedetta.tugnoli@vetagro.com; 2 Dipartimento di Scienze Mediche Veterinarie, DIMEVET-Università di Bologna-Via Tolara di sopra, 50-40064 Ozzano Emilia, Bologna, Italy; giulia.giovagnoni4@unibo.it (G.G.); ester.grilli@unibo.it (E.G.); 3 Vetagro Inc., 116 W. Jackson Blvd., Suite #320, Chicago, IL 60604, USA

**Keywords:** organic acids, pig, acidifiers, metabolic effect, microencapsulation

## Abstract

**Simple Summary:**

Organic acids have been used for a long time to support pig growth particularly at weaning, and more recently have become the number one alternative to growth promoters to improve the production efficiency of pigs. This article will review the antimicrobial properties of organic acids and elucidate the different modes of action that organic acids can exert along the gastrointestinal tract of pigs. Moreover, it will be introduced the advantage of microencapsulation as a tool to deliver organic acids along the intestine and allow their positive effects.

**Abstract:**

Organic acids have been used successfully in pig production as a cost-effective performance-enhancing option and they continue to be the number one alternative to antibiotic growth promoters. The aim of this review is to provide the biological rationale behind organic acids use in pig production, focusing on their different effects along the gastrointestinal tract of pigs. Organic acids are reviewed for their antimicrobial properties and for their classic use as acidifiers, with particular attention to pH modulation and microflora control. Additional beneficial effects on intestinal health and general metabolism are presented and we explain the advantage of microencapsulation as a tool to deliver organic acids along the intestine.

## 1. Introduction

Today the modern livestock industry requests increasingly high animal productivity and efficiency. In the past, high levels of production were obtained using pharmaceutical strategies such as antibiotic growth promoters. Health concerns have driven worldwide regulatory restrictions, limiting the use of antibiotics and favoring the diffusion of new types of production (such as antibiotic-free and no antibiotic ever) whose feasibility requires the highest standards of management, feeding, and nutrition. To support animal growth and health without drugs, diet has a pivotal role beyond the supply of nutrients, and high quality feed ingredients and feed additives are therefore needed. Among them, organic acids have been widely used over the last decades for their positive effect on growth efficiency and they are considered a valid tool for pig production. Organic acids were reported as effective growth enhancers throughout the production cycle of pigs, although there is a relatively large variation in responses due to various factors such as type and dose of organic acids used, supplementation duration, type of diet and buffering capacity, hygiene and welfare standards, health status, and age of the animals [1,2]. The magnitude of growth response was shown to be greater in weaning pigs than in older animals. For example, in a meta-analysis study, conducted by Tung and Pettigrew, the improvements of growth rates were 12.25% and 6.03% for the first 2 weeks or 4 weeks post-weaning respectively, while the enhancement was lower for growing (3.51%) or finishing (2.69%) pigs [2].

Even with a level of variability, all the literature available suggests that organic acids effects on pig growth performance are consistent, thus supporting their use in practical conditions. From this starting point, the aim of the present work is to provide the biological rationale behind the use of organic acids in pig nutrition, focusing on the different mode of action they can exert in the diet and then travelling along the gastro-intestinal tract of pigs.

## 2. Classification of Organic Acids

Organic acids can be classified in three main functional categories: short chain fatty acids (SCFA), medium chain fatty acids (MCFA), and tricarboxylic acids (TCA) [3].

SCFA are carboxylic acids with max 5 carbon atoms and they are produced in the lower intestine of animals by the microbial fermentation of indigestible sugars and amino acids. In particular, acetic, propionic, and butyric acid are produced in a physiological ratio (60:25:15), having a key role for intestinal mucosa trophism and general metabolism [4,5]. In swine and piglets, they improve intestinal morphology and have a beneficial effect on the intestinal barrier, decreasing intestinal inflammation [6,7]. Thanks to their liquid status, SCFA are mostly used as feed acidifiers, silage inoculants, and preservatives in animal nutrition.

MCFA have aliphatic chains with 6 to 12 carbon atoms. These acids can be quickly incorporated in the membrane of phospholipids and they play a relevant role in the nutrition of young piglets, where they represent an important energy source [8,9]. Thanks to their high pK_a_ MCFA have a higher antibacterial activity compared to SCFA in the hindgut [10].

TCA are metabolic intermediates of Krebs cycle, thus involved in energy metabolism. These acids improve gut morphology and barrier function, with a favorable influence on microbiota [11,12].

Other than these categories, there are a few organic acids like benzoic, sorbic, and lactic acid that are widely used in food and feed preservation thanks to their antifungal and antimould properties.

When managing organic acids in animal nutrition there are several properties to consider, i.e., physical form, flavoring properties like odor and taste, solubility in water and also the safety aspect. Table 1 shows the list of the most common organic acids and their properties, grouped by the aforementioned categories. Organic acids with the highest antimicrobial activity have a pK_a_ between 3 and 5 [13]. It follows that there are different organic acids that can act at different levels on the basis of environmental pH: organic acids with higher pK_a_ can be used with the aim of preserving food/feed, while, from a nutritional point of view, a lower pK_a_ means that the acid acts in the stomach.

## 3. Antimicrobial Properties of Organic Acids

Since many years, organic acids are affirmed as powerful antimicrobials. Their activity against microorganisms is traditionally employed in food preservation, but today their role in animal nutrition has reached the same importance. Organic acids owe their success to a broad spectrum of applications, that are based on antibacterial, antiviral, antifungal, and antimold properties [14,15,16].

Not all organic acids have the same antimicrobial activity. Their action against microorganisms depends on the carbon chain length and degree of unsaturation, but overall the pK_a_ of the acid influences its antimicrobial mechanism of action [17]. Indeed, every organic acid is distinguished by a pH value at which 50% of the acid is found in a dissociated form (pK_a_). In 1998 Russell and Diez-Gonzalez for the first time proposed the anion model, according to which the inhibitory effect of organic acids is highly related to their undissociated form [18]. Based upon the environmental pH and pK_a_ values, organic acids in their undissociated form can diffuse across the bacterial cell membrane and dissociate inside the cell, releasing H^+^ ions and decreasing intracellular pH. To overcome the lowering of pH, microorganisms activate proton pumps consuming energy and, at the same time, the anion RCOO^-^ is toxic to DNA replication, disrupting metabolic functions and increasing osmotic cell pressure [19,20,21]. The combination of these two actions inhibits bacterial replication and growth, leading to bacteriostatic or bactericidal effects. The anion model is generally accepted as mode of action for all the organic acids, but the efficacy of different organic acids can vary mainly upon two factors: on one hand, the lipophilic nature of the acid influences the ability to pass through the microorganism wall; on the other hand, upon dissociation inside the cell, different anions can have different inhibitory mechanisms on cellular functions [22,23,24].

As already mentioned, there are many other variables that affect the antimicrobial activity of organic acids besides the pK_a_. Polar groups, number of double bonds, molecular size, and solubility in non-polar solvents are the four principal chemical and physical features that can predict the inhibitory effect of organic acids, as described by the mathematical model of Principal Components Analysis calculated by Hsiao and Siebert [25].

The spectrum of efficacy of organic acids can vary depending on the nature of the target organism and, in particular, on the complexity and structure of its outer cell wall and/or membrane [26]. Gram positive bacteria (i.e., *Clostridium perfringens, Enterococcus spp., Streptococcus spp.)* are mainly susceptible to MCFA, while Gram-negative bacteria (i.e., *Escherichia coli, Campylobacter jejuni, Salmonella spp.*) are more sensitive to SCFA [27,28]. This can be explained by the lipophilic nature of MCFA that allows them to have a stronger antibacterial activity mainly against Gram-positive species, whereas the presence of lipopolysaccharide (LPS) in the Gram-negative cell wall confers resistance to these species [29]. Propionic acid and butyric acid are strong mold inhibitors, while acetic acid is commonly used as antifungal, also reducing aflatoxins production [30,31,32].

## 4. Targeting the Feed and the Stomach

In pig nutrition, organic acids are traditionally used as “acidifiers” with two targets, the feed and the stomach where the main aim is to reduce the pH.

### 4.1. Effects in the Feed

Organic acids are included in the feed to prevent spoilage and improve the hygienic quality, with particular reference to undesirable molds and bacterial growth inhibition. For this purpose, in the European Union several organic acids such as formic and propionic acids, as well as others (lactic, citric, fumaric, and sorbic acid) and their salts (e.g., calcium formate, calcium propionate) are authorized as feed additives in the category named “feed preservatives” [33]. A great variety of molds inhibitors are available on the market, from single acids in liquid form to blends of different organic acids and their salts on carriers, with different applications. In general, buffered products such as acids salts are preferred over acids for their higher ease in handling and lower corrosiveness [34]. Beside the antimold effects, organic acids can act as antimicrobials primarily by reducing the pH of the feed, so altering the growth conditions for microorganisms, and secondly by a direct inhibition on the growth of specific bacteria. The effect of organic acids on diet pH has been thoroughly reviewed by Tung and Pettigrew that, in a meta-analysis, showed an appreciable reduction of diet pH (from 5.95 to 4.71) due to the addition of acids [2].

Moreover, organic acids can help in reducing the growth of specific pathogenic bacteria (such as *Salmonella spp.*) in the feed and therefore the risk of microbial contamination for the animal, gaining importance from a food safety perspective. In this regard, several studies investigated the effects of acids either as individual components or as mixtures of acids on feed materials artificially contaminated with *Salmonella*. The efficacy of organic acids in reducing *Salmonella* counts was variable upon various factors such as the type of acid, the nature of acid (free or salt), the inclusion dose, the bacterial load, and the feed ingredients. Interestingly, the lowest and slowest reduction of *Salmonella* with acidification was observed in soybean meal (47.8% of crude protein), suggesting a protective effect on *Salmonella* cells for protein-rich matrices [35].

### 4.2. Effects in the Stomach

At the gastric level, the primary goal of organic acids is the reduction of pH, key-point particularly for young, newly-weaned animals. At this age the gastro-intestinal tract is not fully developed and stomach pH tends to be high (often over 5) due to a combination of poor endogenous HCl secretion, lack of lactic acid from lactose fermentation, and intake of large meals at infrequent intervals [6]. High pH ultimately impairs pepsin activation and function, that is optimal in acidic environment at pH 2 to 3.5, therefore strongly reducing the efficiency of protein digestion [36]. There is also evidence that high pH also increases the rate of gastric emptying thus reducing the time for the feed to be digested in the stomach [37]. Beside these physiological aspects, gastric pH of weaning piglets is kept high also by the high buffering capacity of the diet, particularly determined by some ingredients such as milk products, vegetable proteins, and meat/fish meal, so that acidification can help the transition from milk to solid feed. Indeed, to overcome all these issues related to high pH, the inclusion of organic acids is aimed at lowering stomach pH.

However, data showed inconsistent results about gastric pH modulation: while some studies reported a significant reduction, others found no effects on gastric pH following dietary acidification. In a meta-analysis collecting data from 22 studies using individual organic acids at high doses (1% to 2%), stomach pH was lower in 55% of the cases, higher in 36% and equal in 9% of the cases for acidified diets compared to control [2]. More recently, no relevant effects on gastric pH were reported for different combinations of organic acids, mainly fumaric acid-based blends, MCFA, or mixtures of organic acids and MCFA fed to weaning pigs [38,39,40,41]. In a study conducted by Zentek and coworkers, a fumaric and lactic acid mixture did not have any effect, whereas a blend of caprylic and capric acid reduced pH in pylorus, but not in cardia and fundus of the stomach [42] These inconsistencies among different studies may be due to the fact that gastric contents are heterogeneous, and that there are different pH values in different stomach regions. In addition, the time of sampling can have an effect on pH values as gastric pH is subjected to fluctuations over time after feeding [43]. Interestingly, formic acid fed at 1.8% to weaning pigs kept gastric pH at values below 3 for several time-points after feeding, in particular it prevented the physiological rise in pH observed in control animals after the meal. However, when considering the mean of all sampling times from 0.5 to 8.5 h post-feeding, the gastric pH value was not affected by formic acid [44]. This result shows that formic acid at high doses can help in counteract the buffering capacity of the diet but, at the same time, it suggests that sampling time can often explain the different outcome between different studies.

Although with inconsistent effects on gastric pH, organic acids were shown to positively affect nutrient digestibility especially fecal dry matter (+0.82%) and crude protein digestibility (+1.33%) with the response being variable upon acid type, acid dose, and diet type. Improvements were generally higher for diets formulated with plant and animal origin feed ingredients compared to only plant-based diets [2]. Regarding MCFA, caprylic and capric acids, both individually and in combination, increased total tract protein and fiber digestibility in weaning pigs [39]. Other studies did not observe a beneficial effect on nutrient digestibility (either ileal or total tract) by supplementing weaning diets with either fumaric, formic, or lactic acid individually or with mixtures of various organic acids [45,46,47]. It is noteworthy that fumaric acid, included to a diet with low buffering capacity, increased the ileal digestibility of crude protein, gross energy and the majority of amino acids, but these beneficial effects were reduced in case of a high buffering capacity diet [48]. Regarding growing pigs, positive effects on the ileal digestibility of several amino acids have been reported for propionic and lactic acid, whereas commercial blends of organic acids and MCFA showed higher apparent total tract digestibility of gross energy, dry matter, organic matter, and crude fiber without affecting amino acids ileal digestibility [49,50,51]. It is important to point out that these digestibility trials, both ileal or total tract, provide estimates of the amount of nutrients digested and absorbed within the GIT, therefore the positive impact of organic acids can be explained by a promotion of either gastric digestive function or intestinal absorption, two aspects not easily discernible analytically.

In addition, various organic acids (such as citric, formic, fumaric, and lactic acid) may improve the absorption and retention of some minerals including calcium, phosphorus, magnesium, and zinc, thus increasing their biological value and limiting their excretion. More precisely, citric acid seems to increase calcium and phosphorus absorption, likely by chelating calcium and making the phytate structure less stable and more accessible to phytase action [2,52,53].

Low pH in the stomach is beneficial for proper enzymatic activities and protein digestion but, at the same time, can select acid tolerant undesirable microorganisms. Bearson et al. reviewed that enterobacteria (including *S. typhimurium* and *E. coli*) have evolved inducible acid survival strategies to face acid stress conditions characterized by low pH and high concentration of weak organic acids [54]. These systems include the activation of acid shock proteins and inducible decarboxylases that consume protons for decarboxylation reactions and exchange end-products for new substrates thanks to membrane transporters [55,56]. Furthermore, it has been shown that some SCFA (such as formic and acetic acid) may serve as environmental signals to trigger the expression of invasion genes in *Salmonella* [57,58]. Therefore, an early exposure of pathogens to acidic conditions in the stomach can prepare them to endure stress conditions more distally in the small intestine and intensify their pathogenic action.

## 5. Targeting the Intestine

Beyond the stomach, organic acids are meant to work along the intestine where most of the microbial colonization/proliferation occur and their antimicrobial action is needed. In addition, organic acids can have a metabolic effect serving as energy source for the intestinal mucosa and overall modulate general metabolism.

### 5.1. Modulation of Microflora

Based on their antimicrobial mode of action, organic acids are able to inhibit the growth of undesired pH-sensitive microorganisms like *Enterobacteriaceae*, while they do not affect beneficial lactic acid bacteria. In this view, organic acids can be an optimal tool to control the dysbiosis characterized by coliforms overgrowth and *Lactobacilli* depression, typical of weaning [59]. Many studies investigated the effects of dietary acidifiers on microbial populations along the digestive tract of weaning piglets with dissimilar results, mainly dependent on the acid used (type and dose) and the intestinal tract analyzed. For example, Grecco et al. reported that fumaric acid at 0.8% reduced total coliforms and *E. coli* numbers in the cecum of weaning pigs, whereas in other trials no effects on microflora all along the gastro-intestinal tract were observed with 1.5% fumaric acid [40,60]. Also citric acid at 1.5% did not show significant effects on microflora populations (total anaerobes, *Lactobacilli*, *Clostridia*, *E. coli*) or incidence of scouring after an *E. coli* challenge in weaning pigs [60,61]. However, in weaned pig challenged with both *S. typhimurium* and *E. coli*, 0.5% of citric acid decreased both *Salmonella* and *E. coli* counts in fecal samples, while increasing *Lactobacilli* number [62]. Formic acid included at doses ≥1%, either as pure acid or salts, was generally effective in decreasing coliforms along the gastro-intestinal tract, being the microbial changes more evident in the small intestine than in the lower gut in some cases [44,63,64,65,66]. These inconsistent effects along the GIT are likely related to the lack of sufficient amounts of undissociated acids after the stomach, finally impairing organic acids antimicrobial action. Indeed, in many cases organic acids supplemented with the diet were recovered at concentrations higher than unsupplemented control only in the stomach and in the proximal small intestine then disappeared in the distal small intestine and large intestine contents [42,60,67,68]. Regarding MCFA, caprylic acid, and to a lesser extent capric acid at 0.3%, allowed to reduce *E. coli* counts both in jejunum and cecum digesta of weaning pigs [69]. Blends of organic acids and MCFA showed variable results either reducing *E. coli* and increasing microbial diversity in the colon or not affecting at all large intestine or fecal microflora [40,41,70].

### 5.2. Modulation of Fermentation Patterns

Organic acids have been studied not only for their antimicrobial activity against specific bacterial strains, but also for their ability to modulate intestinal fermentation patterns. Some studies from our research group evaluated the effects of different organic acids on microbial growth and ammonia production by pig cecal microflora, using an in vitro fermentation system with fresh cecal content as bacterial inoculum. Various organic acids were tested (among which SCFA, Krebs cycle acids, and preservatives) showing that different types and concentrations of organic acids can either inhibit (e.g., sorbic, benzoic, formic acid) or enhance (e.g., lactic, citric, fumaric acid) the bacterial fermentation, at the same time reducing ammonia production (particularly for sorbic, benzoic, and alpha-ketoglutaric acid) [71]. Furthermore, in the same system, a blend of Krebs cycle acids (citric, fumaric, malic acid plus phosphoric acid) was effective in reducing both ammonia and the concentration of iso-acids such as iso-butyric and iso-valeric, thus indicating a strong reduction of microbial proteolysis, at the same time reducing also cellulolytic activity as shown by lower acetic acid levels [72]. Partanen and Jalava developed a different in vitro fermentation model using frozen ileal digesta plus fresh fecal samples as bacterial inoculum: among others, formic acid, potassium sorbate, and sodium benzoate were the most effective in modulating bacterial fermentation, by reducing total volatile fatty acids (VFA), acetic acid, and propionic acid concentrations compared to control, although not affecting ammonia levels [73]. It is noteworthy to point out that these in vitro studies are strictly dependent on the bacterial inoculum and its source (intestinal tract vs. fecal, fresh vs. frozen) that can likely explain some different final outcomes. Moreover, as additional drawback, the batch fermentation set-up differs from in vivo conditions primarily because microbial end-products accumulate in the medium due to lacking physiologic digesta flow and mucosa absorption. However, such systems can be useful to screen the effects of potential feed additives, among which organic acids, on intestinal fermentation patterns or to assess the fermentability of feed ingredients before in vivo studies [74].

In vivo, the effects of dietary organic acids on fermentation patterns along the intestine appeared to be more variable, as previously reviewed [6,52]. Formic acid was one of the most studied and in some cases doses in the range of 0.3% to 3% did not alter ammonia and VFA production along the GIT [75,76,77]. However, in other studies formic acid or its salts increased acetic acid and decreased lactic acid concentrations in both ileum and cecum-colon contents [44,68,78,79]. These findings can indicate a shift in the composition of intestinal flora and a modulation of microbial fermentations with more nutrients (like glucose not fermented into lactic acid) or metabolites (like acetate) available to the host [79]. Supplementation of propionic acid-based products to weaning pigs’ diets showed inconsistent effects, generally not affecting pH, ammonia, SCFA, and microflora populations along GIT, but sometimes decreasing *E. coli* counts in the stomach and increasing *Lactobacilli* number in the duodenum compared to controls [77,80,81].

Concerning Krebs cycle acids, the addition of either citric or fumaric acid at 1.5% did not affect pH, VFA concentrations, or microflora populations along the GIT of weaning pigs [60,61]. Zentek and colleagues investigated the effects of combinations of organic acids (fumaric and lactic acid) and MCFA (capric and caprylic acid) fed to weaning piglets finding only minor changes on the gastro-intestinal ecology and microbial metabolites production. Nevertheless, in the colon digesta an interesting reduction of some *E. coli* pathogenicity factors was observed particularly for organic acids that may be relevant to support intestinal health at weaning [42]. More recently, different commercial blends of organic acids including SCFA, MCFA, and acids salts were shown to increase the levels of acetic, propionic, and butyric acid produced by microbial fermentation of carbohydrates in the large intestine, while reducing *E. coli* counts [41]. In addition, other mixtures of organic acids (including MCFA) fed to growing pigs increased VFA content in the ileum and fiber fecal digestibility, indicating a positive stimulation on microflora that can utilize carbohydrates in the large intestine and produce SCFA [51].

Overall, organic acids have the potential to modulate the microflora populations and consequently microbial metabolites production along the GIT. The modulation of VFA can be of particular interest because of their pivotal role for the intestinal physiology and metabolism, as presented in the following paragraph. Nevertheless, these results appear to be often inconsistent and it is quite difficult to compare the experimental studies because of the employment of different organic acids (pure acids or salts, individually or in variable combinations) but also because of additional factors such as the intestinal tract analyzed and, among all, the diet composition. Furthermore, organic acids frequently appear as not effective, even when they are used at high doses, likely due to their variable ability to reach the intestinal sites at sufficient levels for early dissociation or absorption in the stomach.

### 5.3. Metabolic Effect-Trophic Factors

A metabolic role in the intestinal tract can be notably attributed to SCFA (mainly acetic, propionic, and butyric acid) produced by microbial fermentations and physiologically relevant for the large intestine, but also to TCA involved in Krebs cycle.

#### 5.3.1. Short Chain Fatty Acids

SCFA are end-products of microbial fermentations of carbohydrates occurring mostly in the large intestine of hindgut fermenter animals. In the large intestine of pigs, SCFA are usually found at a molar ratio of approximately 60:25:15 for acetic:propionic:butyric and they are rapidly absorbed by the colonic epithelium to play a pivotal role in the intestinal and general energy metabolism. Butyric acid is almost completely oxidized within the mucosa, serving as preferred energy source for the colonocytes, whereas propionic acid is collected by the liver where it is converted into glucose, and acetic acid is instead used by peripheral tissues [4,82].

The metabolic effects of supplementing diets for pigs with SCFA have been variably investigated. Literature about propionic and acetic acid in pigs is quite scarce although a possible role for acetic acid in improving protein metabolism through a nitrogen-sparing effect was suggested in a single study with growing pigs but then not confirmed under practical conditions [83,84]. For butyric acid most of the attention was given to its impact on the intestinal mucosa, not only for its role as trophic factor but also for its multi-functional properties such as anti-inflammatory, anti-oxidant and protective for the epithelial integrity that can be useful to enhance intestinal health [85,86,87]. Addition of butyric acid to diets for weaning piglets has been reported to positively affect the gut morphology, favor gastric mucosa development, reduce the inflammatory stress and improve immune status and intestinal barrier function [88,89,90,91]. However, the final outcome in terms of growth performance for dietary butyric acids was not always consistent and highly dependent on several factors such as dose, supplementation duration, and age of piglets [92]. Other studies investigated the impact of butyric acid triglyceride, named tributyrin, as a tool to provide more butyric acid and enhance its effects. Dietary tributyrin combined with lactitol, a fermentable source of butyrate, improved the intestinal trophism of weaning piglets, as indicated by longer villi, shorter crypts, higher polyamines concentration and increased intestinal enzymes activities [93,94,95]. Additionally, tributyrin was particularly effective in supporting the intestinal development and growth of intrauterine growth restriction (IGR) piglets namely piglets born with lower body weight than normal neonates. Tributyrin not only improved intestinal morphology, barrier function, and digestive activities but also attenuated lipid metabolism dysfunctions usually associated with IGR piglets, thus suggesting a role for TB in modulating general metabolism [96,97]. In this line, recent studies by Murray and colleagues demonstrated that tributyrin fed to neonatal piglets can promote muscle growth by directly increasing the myogenic potential of muscle satellite cells, therefore appearing a promising nutritional tool for proper muscle development and pig growth [98].

Overall, dietary SCFA and especially butyric acid can exert a metabolic effect not limited to the intestinal site but also involving general metabolism, with positive consequences on health and growth.

#### 5.3.2. Krebs Cycle Acids

TCA involved in Krebs cycle contribute to energy metabolism by definition. Some of them, such as citric and fumaric acid, are commonly used in pig nutrition as acidifiers and it is interesting to understand whether they can actually have a metabolic role at the intestinal level beyond their acidic properties.

The intestinal absorption of citric and fumaric acid was studied using brush border membrane vesicles isolated from pig proximal jejunum: citric and fumaric acid uptake was shown to occur through a Na-dependent co-transporter that is common for other TCA [99]. As demonstrated in hamster small intestine, TCA following the uptake at the apical side are metabolized inside the enterocyte and then leave the basolateral side partly as such and partly as metabolites. The extent of metabolism inside the cell appeared to be 60%–70% of the absorbed acid, with a 30%–40% effectively being transported to the serosa side [100]. More recently, studies with pigs showed that alpha-ketoglutaric acid (AKG) is better absorbed in the small intestine than in distal sites, then quickly metabolized in the enterocytes and liver so that half-life in blood is very short [101,102]. These results provide evidence that dietary Krebs cycle acids can be metabolized within the enterocytes, therefore directly influencing intestinal metabolic status. It has been proposed that the local trophic effect on the small intestine mucosa can explain the positive effects of fumaric acid in terms of improved digestive and absorptive functions [48]. Moreover, Kirchgessner and Roth suggested that ingested fumarate is metabolized in Krebs cycle as well as fumaric acid produced by metabolic routes and, additionally, pigs can utilize it as energy source with an efficiency close to that of glucose [103]. More recently, succinate was shown to improve intestinal morphometry (longer villi, shorter crypts) in growing pigs and to enhance the barrier functionality of pig intestinal cells in vitro, by increasing tight junction markers’ expression [12].

Beyond the intestine, the effects of dietary supplementation with citric and fumaric acid have been studied mainly on intermediary liver metabolism in rats: the activity of Krebs cycle enzymes was generally not affected but liver transaminases (glutamate dehydrogenase, glutamate oxaloacetate transaminase, and glutamate pyruvate transaminase) were increased following citric/fumaric acid supplementation, suggesting that their carbon skeleton could have been used to synthesize non-essential amino acids [84].

Alpha-ketoglutaric acid is very attractive for its multiple role in cell metabolism, especially as bridge between energy and protein metabolism, but also precursor for the synthesis of glutamate and glutamine [104]. The specific role of AKG at the intestinal level has been already thoroughly reviewed with particular reference to its positive effects on intestinal morphology, anti-oxidative capacity, and absorptive and barrier function [105,106]. In weaning piglets challenged with *E. coli* LPS, dietary AKG supplementation improved both small intestine and liver morphology and function thus ameliorating the endotoxin-induced injury [107,108,109]. Moreover, the addition of AKG to a low-protein diet in pigs allowed to improve nitrogen, Ca and P metabolism and promote both protein and lipid metabolism in skeletal muscle (favoring intramuscular fat and monounsaturated fatty acids content), thus being a promising nutritional strategy to support pig productivity and supply high-quality pork [110,111,112].

#### 5.3.3. Others

Also, sorbic and benzoic acid, commonly used as feed preservatives for their antimicrobial properties, can modulate general metabolism with different modes of action.

Sorbic acid is metabolized in the body like other fatty acids, being subject to beta-oxidation and serving as energy source. Studies with murine models showed that sorbic acid is rapidly absorbed and completely oxidized to H_2_O and CO_2_ [113,114]. In addition, sorbic acid added to weaning piglets increased growth performance through the modulation of lipid metabolism and the enhancement of insulin-like growth factor system, particularly relevant for the GIT development [115,116].

On the other hand, benzoic acid is almost completely excreted in urine as its glycine conjugate, hippuric acid, in pigs [117]. Benzoic acid has been reported to reduce urine pH and ammonia emissions from pig slurry, thus with positive consequences in terms of environmental impact of pig production in commercial settings [118,119].

## 6. Use of Protected vs. Free Organic Acids

In the last decade, the use of organic acids “protected” using different coating and microencapsulation techniques has become well established. The protection of organic acids is of great interest for the pig nutrition industry because it allows various technological advantages: it improves handling and safety, increases stability, reduces dustiness and corrosiveness problems, and prevents damages due to temperature/pressure during processing, as well as undesirable interactions with other ingredients [120]. Moreover, microencapsulation allows to increase palatability by masking unpleasant smell/taste and, even more importantly, to deliver the ingredients to specific target sites of action inside the animal, providing therefore also “biological” advantages.

Studies from our research group demonstrated that lipid microencapsulation of organic acids (i.e., the inclusion in a lipid matrix) prevents the rapid disappearance of organic acids right after the stomach and, conversely, allows them to be slowly released along the small and large intestine of pigs, thanks to the action of intestinal lipases [121]. As a consequence of their targeted delivery, lipid encapsulated organic acids were effective in exerting their antimicrobial action along the intestine, by reducing coliforms counts both in distal jejunum and cecum, whereas free organic acids did not have any effect [121]. Additionally, comparing free and protected organic acids in weaned pigs, lipid microencapsulation allowed to reduce the effective dose of organic acids included in the diet by 10 fold compared to the non-encapsulated organic acids, still maintaining the same performance results [122].

Currently, the feed additive industry offers a wide range of organic acids-based products, protected or encapsulated in different forms, containing either individual organic acids, mixtures of organic acids, or blends of organic acids coupled with other active compounds. Particularly interesting and efficacious is the combination of organic acids with botanicals, as they can interact positively and exert antimicrobial properties in a synergistic way: botanical aromatic compounds, as pore-forming agents, can alter the bacterial membrane, eventually facilitating the organic acids entrance and therefore the antimicrobial action [3]. Some in vitro studies demonstrated the synergy between organic acids and pure botanicals, showing that their combination is more effective than each compound alone against food-safety related bacteria such as *S. typhimurium* and *C. jejuni* [3,123]. Again, a combination of organic acids and botanicals in pigs was effective in reducing *S. typhimurium* shedding throughout a production cycle from weaning to slaughter [124].

Beside the promising application of organic acids and botanicals for foodborne pathogens control, selected combinations of organic acids such as citric and sorbic acid together with monoterpenes were shown to exert a direct effect on the intestinal mucosa, by improving the barrier integrity of Caco-2 intestinal cells, without the mediation of the microflora. When these active ingredients were encapsulated in a lipid matrix and fed to weaning pigs, improved barrier functionality in jejunum and ileum and reduced local and systemic inflammatory pressure were observed and then translated in better growth performance [125].

Without claiming to be exhaustive, the above-mentioned examples suggest that microencapsulation can confer an additional value to organic acids and that the combination with other bioactive molecules such as botanicals can further enhance their antimicrobial action and allow a general intestinal health-promoting effect.

## 7. Conclusions

Based on the literature review, the key points about organic acids in pig nutrition can be summarized as follows:

- The positive effects on growth performance are consistent and largely supported by the extensive use of organic acids in practical conditions. Instead, the impact in terms of reduction of gastric pH, improvement of protein digestibility, and modulation of microflora is more variable and generally achieved including organic acids in the diet at high doses (above 1%).
- Most of the published studies are quite dated and involve the use of formic, fumaric, and citric acid reflecting their use as feed additives, well-established, over the years. More recently, combinations of organic acids and MCFA have been thoroughly tested and proposed on the market with positive results.
- More than acidifiers, organic acids can have a metabolic role, by improving the intestinal mucosa trophism and modulating general metabolism, particularly for SCFA and Krebs cycle acids. Butyric acid-based products have a strong history of use, while alpha-ketoglutaric acid is very attractive for future applications.
- Protecting organic acids with encapsulation techniques can provide both technological and biological advantages, target-delivering organic acids along the intestine where they can exert their beneficial effects.

In conclusion, organic acids have multiple properties that can be useful to support health and growth of pigs and they continue to be pivotal feed additives in pig nutrition.

## Figures and Tables

**Table 1:** List of the most common acids and their properties.

Category	Acid	Molecular Formula	pK_a_	Physical Form	Odor and Taste	Solubility	Chemical Safety
**Short Chain**	Formic	CH_2_O_2_	3.75	Colorless liquid	Pungent odor	Miscible in water, ether, acetone, ethyl acetate, methanol, ethanol	Severe skin burns and eye damage
	Acetic	C_2_H_4_O_2_	4.74	Colorless liquid	Pungent vinegar-like odor Sour and burning taste	Miscible in water, alcohol, glycerol, ether, carbon tetrachloride	Flammable liquid and vapor, severe skin burns and eye damage
	Propionic	C_3_H_6_O_2_	4.88	Colorless liquid oily	Very pungent rancid odor	Miscible in water, soluble in alcohol, ether, chloroform	Severe skin burns and eye damage
	Butyric	C_4_H_8_O_2_	4.82	Colorless liquid oily	Rancid unpleasant odor, acrid taste, with a sweetish after taste	Miscible in water, alcohol, ether	Severe skin burns and eye damage
**Medium Chain**	Caproic (or hexanoic)	C_6_H_12_O_2_	5.09	Colorless to light-yellow liquid oily	Characteristic goat-like odor	Soluble in ethanol and ether	Toxic in contact with skin, severe skin burns and eye damage
	Caprylic (or octanoic)	C_8_H_16_O_2_	4.89	Colorless to light-yellow liquid oily	Characteristic goat-like odor	Miscible in ethanol, chloroform, acetonitrile Soluble in alcohol, chloroform, ether, carbon disulfide petroleum ether and glacial acetic acid	Severe skin burns and eye damage; harmful to aquatic life with long lasting effects
	Capric (or decanoic)	C_10_H_20_O_2_	4.90	White crystalline powder	Characteristic goat-like odor	Soluble in ethanol, alcohol, ether, chloroform, benzene and carbon disulfide	Skin and eye irritation; harmful to aquatic life with long lasting effects
	Lauric (or dodecanoic)	C_12_H_24_O_2_	5.30	White flakes	Bay-like odor	Miscible with benzene Very soluble in methanol and ethanol Soluble in acetone	Skin irritation, serious eye damage and irritation
***TCA***	Citric	C_6_H_8_O_7_	3.13 4.76 6.49	White or colorless crystalline powder	Odorless Pleasant and sour taste	Very soluble in water and ethanol Soluble in ether and ethyl acetate	Skin irritation, serious eye damage and irritation
	α-ketoglutaric	C_5_H_6_O_5_	2.47 4.68	White crystalline powder	Odorless	Soluble in water and alcohol	Skin irritation, serious eye damage. May cause respiratory irritation
	Fumaric	C_4_H_4_O_4_	3.02 4.76	White crystalline powder	Odorless Fruit-like taste	Soluble in ethanol and concentrated sulfuric acid	Serious eye irritation
	Malic	C_4_H_6_O_5_	3.40 5.10	Liquid or white crystalline powder	Odorless Apple or tart taste	Very soluble in methanol, ethanol, acetone, ether, and other polar solvents	Harmful if swallowed; skin and eye irritation. May cause respiratory irritation
**Others**	Sorbic	C_6_H_8_O_2_	4.76	White crystalline powder or granules	Odorless Acrid and sour taste	Very soluble in ether. Soluble in ethanol	Skin and eye irritation. May cause respiratory irritation
	Benzoic	C_7_H_6_O_2_	4.19	Colorless crystalline powder	Pungent odor Bitter taste	Soluble in alcohol, ether, and benzene	Skin irritation, eye damage; damage to organs through prolonged or repeated exposure
	Lactic	C_3_H_6_O_3_	3.86	Colorless to yellow viscous liquid or crystals	Odorless Acrid taste	Soluble in water, ethanol and diethyl ether	Skin irritation, serious eye damage
